# Surgical Resection of a Giant Hepatic Hemangioma in a Hepatitis B-positive Patient: A Case Report

**DOI:** 10.7759/cureus.65502

**Published:** 2024-07-27

**Authors:** Nikhil Thatipalli, Rajesh G Gattani, Sarika Peddi, Shakti Sagar, Bhagyesh Sapkale

**Affiliations:** 1 General Surgery, Jawaharlal Nehru Medical College, Datta Meghe Institute of Higher Education and Research, Wardha, IND; 2 Radiodiagnosis, Nizam's Institute of Medical Sciences, Hyderabad, IND; 3 Pathology, Jawaharlal Nehru Medical College, Datta Meghe Institute of Higher Education and Research, Wardha, IND; 4 Medicine, Jawaharlal Nehru Medical College, Datta Meghe Institute of Higher Education and Research, Wardha, IND

**Keywords:** pedunculated mass, giant hepatic hemangioma, hemangioma hepatitis b-positive, hemangioma, hepatic hemangioma

## Abstract

This case report describes the successful surgical removal of a giant hemangioma in a 41-year-old female with hepatitis B. The patient came with stomach distension, right upper quadrant, and right lumbar region pain. Imaging studies showed a mass measuring 12x7.6x11 cm emerging from the left lobe of the liver, causing compression of surrounding structures. The patient has undergone a laparotomy with successful anatomical resection of the hemangioma. Postoperative recovery was uneventful, and the patient was discharged on the fifth postoperative day. This case highlights the significance of considering surgery for symptomatic giant hemangiomas and normal follow-up to screen for recurrence and aims to present the successful surgical management of a giant hemangioma.

## Introduction

Hemangiomas are a sort of benign tumor characterized by an abnormal overgrowth of blood vessels [1]. The most common type, known as capillary hemangioma, is made up of tiny blood vessels called capillaries [2]. These superficial hemangiomas ordinarily show up during initial stages or early childhood as bright red, raised birthmarks, frequently on the face, scalp, chest, or back [2]. Gratefully, most capillary hemangiomas are safe and go through distinct stages of development followed by gradual shrinkage, eventually vanishing completely by around age five to 10 [3].

In any case, hemangiomas can moreover happen deeper inside the skin or even in inner organs, which are called cavernous hemangiomas, and include bigger blood vessels, giving them a characteristic bluish-purple tone [3]. Whereas cavernous hemangiomas are also benign, their size and location can sometimes cause complications. For instance, a large hemangioma near the eye might reduce vision, or if found close to an organ might possibly interfere with its function [3]. In these cases, treatment options such as medicines (beta-blockers and corticosteroids), laser therapy, or surgery have to be applied to avoid the development of other problems [1,4].

## Case presentation

A 41-year-old female, who is hepatitis B-positive, came to Acharya Vinoba Bhave Rural Hospital (AVBRH) with complaints of abdominal distension and right-sided abdominal and right lumbar region pain for the past six months. The pain was depicted as dull-aching type, without symptoms such as acidity or vomiting. She detailed no critical past medical history, no particular familial history, and no social history involving smoking, drug, or alcohol use.

**Figure 1:**
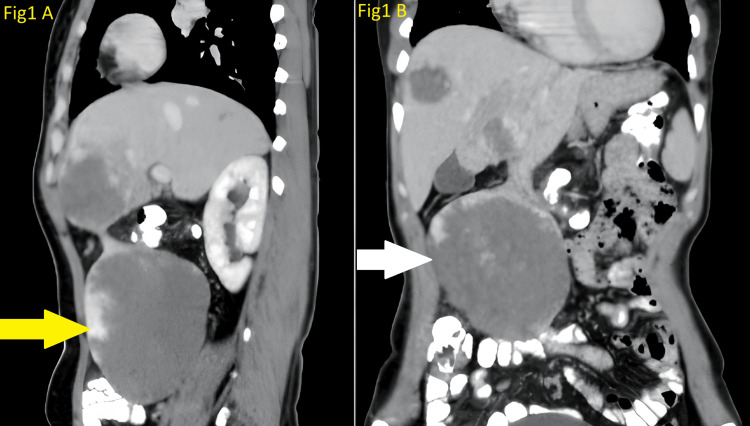
The CECT scan of the patient A: Saggital section view of CECT scan of the patient and a yellow arrow indicating hemangioma; B: Coronal section view of CECT scan of the patient and a white arrow indicating hemangioma CECT, contrast-enhanced computed tomography

On clinical examination, a huge palpable abdominal mass was detected in the right lumbar region, which did not cross the midline. The abdominal ultrasound uncovered a large lesion within the right lumbar region, emerging from the left lobe of the liver, measuring roughly 10.6x10x8 cm. Furthermore, a hemangioma was noted within the right lobe of the liver, measuring 5.5x4 cm. A contrast-enhanced CT scan of the abdomen and pelvis further showed the lesion as an exophytic pedunculated mass emerging from the left lobe (segment 3) of the liver, measuring 12x7.6x11 cm. The contrast-enhanced computed tomography (CECT) scan of the patient is shown in Figure 1.

The lesion showed peripheral nodular enhancement that gradually filled centrally, consistent with a giant hemangioma. It was causing a mass effect, displacing bowel loops, abutting the anterior abdominal wall, and compressing the inferior vena cava and right proximal ureter, leading to minimal hydroureteronephrosis. Three similar lesions were also noted in the right lobe of the liver. We performed surgery to provide symptomatic relief and to prevent torsion of the pedunculated mass in the future. The patient underwent surgery via a large median laparotomy midline incision. The surgical team carefully explored the abdominal cavity to confirm the lesion's location and extent. A huge pedunculated mass was seen arising from the left lobe of the liver. During the surgical procedure, they identified and dissected the pedunculated lesion from the surrounding tissues. The pedicle of the lesion was clamped to control blood flow, and the mass was excised by a bipolar electrosurgical instrument (LigaSure) without any intraoperative incidents. The total blood loss during the procedure was estimated to be around 250 mL. The abdominal cavity was inspected for any residual bleeding or complications before closing the incision in layers. The intra-abdominal drain was placed in the subhepatic space, and the laparotomy incision was carefully closed in layers, with non-absorbable sutures closing the fascia, appropriate approximation of the subcutaneous tissue was done, and staples were used for skin closure.

**Figure 2:**
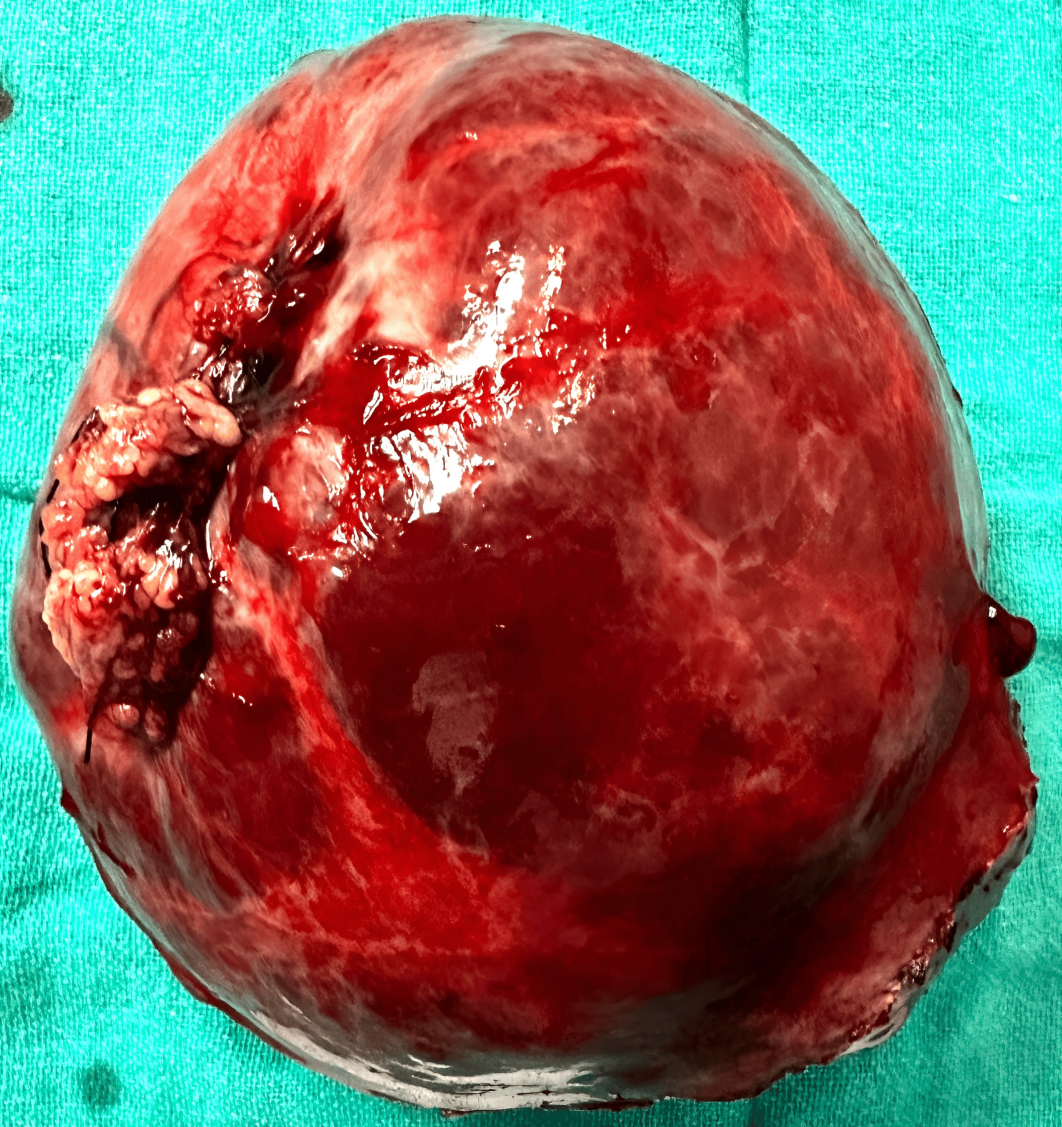
The extracted specimen of the patient

Following surgery, the patient was closely monitored in the recovery room for any anesthesia-related or surgical issues. Postoperatively, broad-spectrum cephalosporin and nitroimidazole antibiotics were started to prevent infections, and the pain was managed effectively with appropriate analgesics. On the second postoperative day, the patient was mobilized out of bed and passed flatus. The patient was permitted a liquid diet followed by a soft diet on the second and third day, respectively. On the third day, the patient passed stools, and the surgical drain was evacuated on postoperative day four. The patient underwent an uneventful postoperative period, with no signs of complications. The patient was discharged on the fifth day, and the removal of skin staples was done on the ensuing follow-up. The patient was prompted for the follow-up to screen for recurrence. The extracted specimen is shown in Figure 2. 

**Figure 3:**
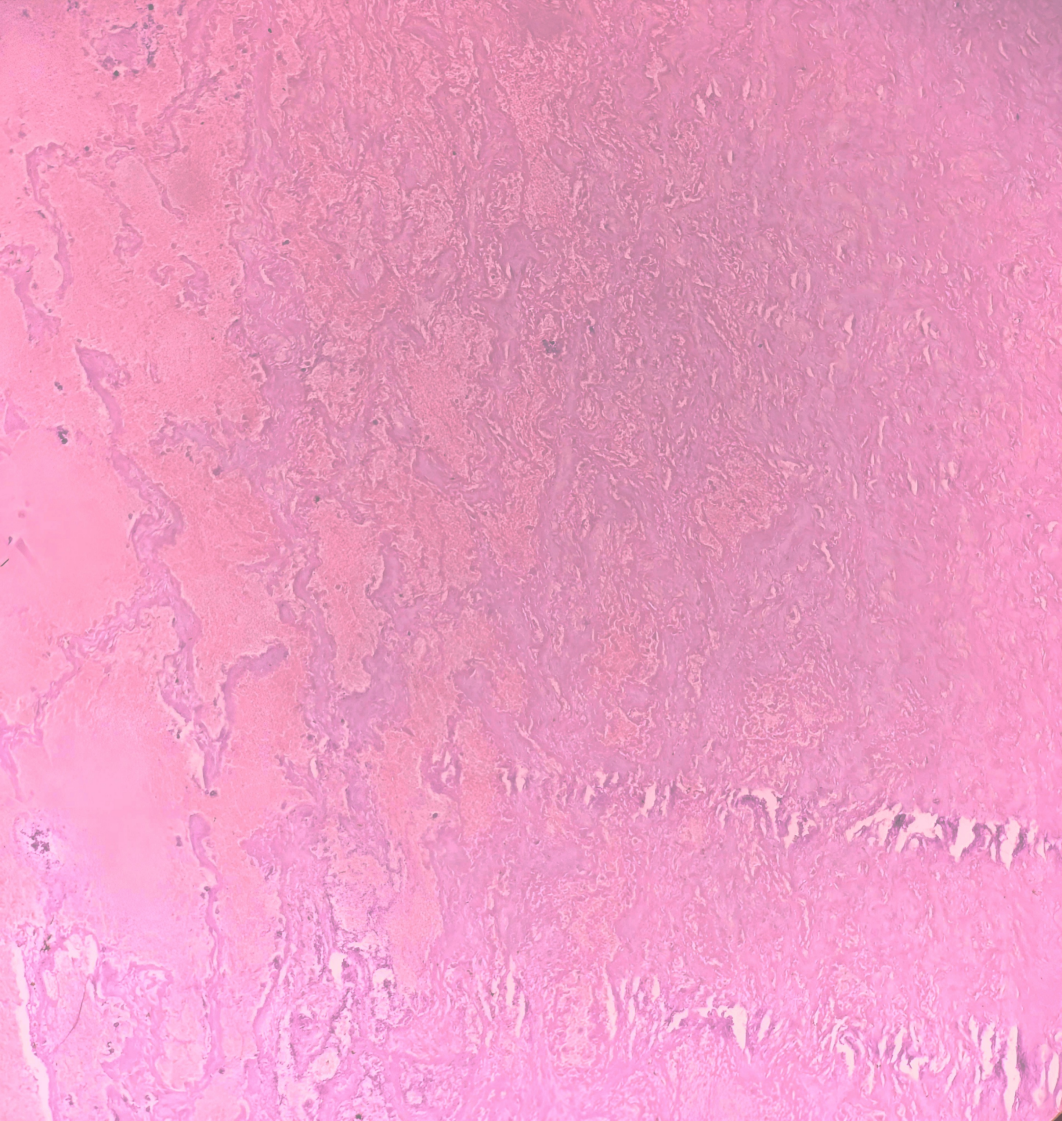
Histopathological image of the patient Circumscribed proliferation of variably sized, dilated, ectatic, and thin-walled vessels lined by a single layer of flat endothelial cells. Irregular interface with surrounding hepatic parenchyma is shown in this figure.

Histopathological examination of the excised specimen confirmed the diagnosis of a giant hemangioma. The histopathological image of the patient is shown in Figure 3.

The successful surgical removal of the lesion alleviated the patient's symptoms, and she was advised to undergo regular follow-up for two years to monitor for the development of new lesions.

## Discussion

We compared the case of our 41-year-old hepatitis B-positive female with a similar case of a 59-year-old female [5]. Both patients presented with abdominal pain, although the 59-year-old female had chronic pain while our patient experienced abdominal distension and right-sided pain for six months, with a palpable mass in the right lumbar region [5]. Ultrasonography and enhanced CT scans were used in both cases. A 59-year-old female had a 4 cm, lobulated mass protruding from the liver, showing progressive peripheral and globular-pattern of a typical hemangioma [5]. In contrast, our 41-year-old had a large exophytic pedunculated mass from the left lobe of the liver, measuring approximately 12x7.6x11 cm, likely a giant atypical hemangioma, along with three smaller lesions in the right lobe. This mass displaced bowel loops, abutted the anterior abdominal wall, and caused minimal hydroureteronephrosis.

Surgical extraction was performed in both cases; however, the approaches changed. The mass within the 59-year-old was evacuated to avoid ischemic complications related to pedicle torsion [5]. In our 41-year-old, a median laparotomy was performed, and the mass was extracted after clamping its pedicle, with negligible blood loss. Postoperative administration for the 41-year-old included broad-spectrum anti-microbials, analgesics, early mobilization, a delicate diet by the third day, and removal of the abdominal drain on the fourth day. She was discharged on the fifth day, and skin staples were removed during follow-up. Pathological investigation affirmed benign hepatic hemangiomas in both cases [5]. The 41-year-old had an uneventful recovery, was symptom-free post-surgery, and was advised to follow-up frequently for two years to screen for recurrence. In outline, whereas both cases include hepatic hemangiomas, they vary in patient background, clinical introduction, mass characteristics, surgical approach, and detailed postoperative care [5]. 

The 52-year-old woman's case detailed by Alharbi LA et al. at King Abdulaziz University Hospital had a history of an expansive hepatic hemangioma since 2011, advancing to incorporate complications such as gigantic ascites and a pulmonary embolism [6]. Despite experiencing radiofrequency removal for symptom relief, her condition weakened, coming about in broad liver association and a new hemangioma within the left hepatic lobe [6]. Laboratory tests showed normal liver function but variations from the norm, indicating a potential coagulation disorder [6]. Imaging affirmed the nearness of a giant hemangioma with liver intrusion and an IVC thrombus. Due to the high-risk nature of surgical resection, she was overseen with medicine and referred for a liver transplant [6].

In contrast, the 41-year-old hepatitis B-positive woman at AVBRH displayed a large palpable abdominal mass, analyzed as a giant hepatic hemangioma. Imaging studies showed different hemangiomas and critical compression of encompassing structures, counting the IVC and right proximal ureter, causing minimal hydroureteronephrosis. Contrasting the 52-year-old woman's case, she underwent effective surgical extraction of the pedunculated mass, which brought about symptom alleviation without intraoperative complications [6]. Her postoperative recovery was uneventful, and she was released on the fifth day with instructions for regular follow-ups to screen for recurrence [6].

The case depicted by Zhou Q et al. includes a 23-year-old primigravida at 37 weeks of gestation, whose fetus was analyzed with congenital hepatic hemangioma (CHH) driving to pulmonary hypertension (PH) due to expanded aspiratory bloodstream from the hepatic vascular shunt [7]. Postnatally, the newborn displayed tachypnea and tachycardia with echocardiographic discoveries characteristic of PH. The treatment included surgical embolization of the hepatic vascular feeders, driving noteworthy enhancement in a patient over a four-month follow-up.

Alternately, our case highlights a hepatitis B-positive female reported with a six-month history of abdominal distension and right-sided stomach pain. Imaging studies showed an expansive exophytic pedunculated mass within the left lobe of the liver and different hemangiomas within the right lobe. The patient underwent surgical extraction of the mass through a median laparotomy, and the postoperative period was uneventful. Pathological examination affirmed a giant hemangioma, and the patient was released with instructions for regular follow-ups to screen for recurrence. Whereas both cases included hepatic hemangiomas, the clinical approaches contrasted altogether, with the primary focus on overseeing life-threatening complications in a neonate, while our case aimed at providing symptomatic relief in an adult through surgical intervention [7].

In June 2016 as reported by Al Farai et al., a 48-year-old Caucasian man displayed loose bowels, vomiting, and epigastric pain exacerbated by coughing or inclining forward, taking after a blunt abdominal injury a month earlier [8]. The clinical examination uncovered a large palpable stomach mass, and imaging studies distinguished a voluminous heterogeneous mass within the epigastric region, suspected to be a gastric submucosal tumor with likely hepatic metastases [8]. In any case, advanced assessment through liver MRI affirmed the determination of a giant pedunculated liver hemangioma starting from the third hepatic segment [8]. Given the hazard of torsion and rupture, surgical extraction was performed without a preoperative biopsy. The patient experienced a left hepatic lobectomy with negligible blood loss, and postoperative recuperation was uneventful [8]. Pathological examination affirmed the diagnosis of hemangioma, with different infarcted or hemorrhagic vascular injuries. The patient remained symptom-free and had no signs of relapse 23 months post-surgery [8].

Also, our case with a 41-year-old hepatitis B-positive woman displayed a six-month history of abdominal distension and right-sided abdominal pain. The clinical examination uncovered a large palpable mass within the right lumbar region. Imaging studies recognized a pedunculated mass from the left lobe (segment 3) of the liver, reliable with a giant atypical hemangioma causing a mass effect. The patient experienced surgical extraction through a median laparotomy. The strategy included cautious dissection and extraction of the pedunculated mass. Postoperative recovery was smooth, with the patient being mobilized and permitted to advance from a fluid to a soft diet by the third postoperative day. Pathological examination affirmed the determination of a giant hemangioma, and the patient was discharged on the fifth postoperative day, with follow-up prompted to screen for recurrence. 

## Conclusions

This case shows the surgical treatment of hemangioma in a 41-year-old female with hepatitis B. The patient displayed stomach distension and right upper quadrant torment. Imaging studies revealed an expansive mass emerging from the left lobe of the liver with an impact on encompassing structures. The patient had a laparotomy with effective resection of the hemangioma. Postoperative recovery was uneventful, and the patient was released on the fifth postoperative day. This case highlights the importance of considering surgery for symptomatic giant hemangiomas. Regular follow-up is done to identify any development of new lesions.
